# Enemy or ally: a genomic approach to elucidate the lifestyle of *Phyllosticta citrichinaensis*

**DOI:** 10.1093/g3journal/jkac061

**Published:** 2022-03-21

**Authors:** Valerie A Buijs, Johannes Z Groenewald, Sajeet Haridas, Kurt M LaButti, Anna Lipzen, Francis M Martin, Kerrie Barry, Igor V Grigoriev, Pedro W Crous, Michael F Seidl

**Affiliations:** Evolutionary Phytopathology, Westerdijk Fungal Biodiversity Institute, Utrecht 3584 CT, The Netherlands; Department of Plant Sciences, Laboratory of Phytopathology, Wageningen University and Research, Wageningen 6708 PB, The Netherlands; Evolutionary Phytopathology, Westerdijk Fungal Biodiversity Institute, Utrecht 3584 CT, The Netherlands; US Department of Energy Joint Genome Institute, Lawrence Berkeley National Laboratory, Berkeley, CA 94720, USA; US Department of Energy Joint Genome Institute, Lawrence Berkeley National Laboratory, Berkeley, CA 94720, USA; US Department of Energy Joint Genome Institute, Lawrence Berkeley National Laboratory, Berkeley, CA 94720, USA; Department of Biology, Institut National de la Recherche Agronomique, UMR INRA-Université de Lorraine “Interaction Arbres/Microorganismes”, Champenoux F-54280, France; US Department of Energy Joint Genome Institute, Lawrence Berkeley National Laboratory, Berkeley, CA 94720, USA; US Department of Energy Joint Genome Institute, Lawrence Berkeley National Laboratory, Berkeley, CA 94720, USA; Department of Plant and Microbial Biology, University of California Berkeley, Berkeley, CA 94720, USA; Evolutionary Phytopathology, Westerdijk Fungal Biodiversity Institute, Utrecht 3584 CT, The Netherlands; Department of Plant Sciences, Laboratory of Phytopathology, Wageningen University and Research, Wageningen 6708 PB, The Netherlands; Theoretical Biology & Bioinformatics, Utrecht University, Utrecht 3584 CH, The Netherlands

**Keywords:** fungal plant pathogens, genomics, CAZymes, lifestyle adaptations, citrus, endophyte, pathogen

## Abstract

Members of the fungal genus *Phyllosticta* can colonize a variety of plant hosts, including several *Citrus* species such as *Citrus sinensis* (orange), *Citrus limon* (lemon), and *Citrus maxima* (pomelo). Some *Phyllosticta* species have the capacity to cause disease, such as Citrus Black Spot, while others have only been observed as endophytes. Thus far, genomic differences underlying lifestyle adaptations of *Phyllosticta* species have not yet been studied. Furthermore, the lifestyle of *Phyllosticta citrichinaensis* is ambiguous, as it has been described as a weak pathogen but Koch’s postulates may not have been established and the presence of this species was never reported to cause any crop or economic losses. Here, we examined the genomic differences between pathogenic and endophytic *Phyllosticta* spp. colonizing *Citrus* and specifically aimed to elucidate the lifestyle of *Phyllosticta citrichinaensis*. We found several genomic differences between species of different lifestyles, including groups of genes that were only present in pathogens or endophytes. We also observed that species, based on their carbohydrate active enzymes, group independent of their phylogenetic association, and this clustering correlated with trophy prediction. *Phyllosticta citrichinaensis* shows an intermediate lifestyle, sharing genomic and phenotypic attributes of both pathogens and endophytes. We thus present the first genomic comparison of multiple citrus-colonizing pathogens and endophytes of the genus *Phyllosticta*, and therefore provide the basis for further comparative studies into the lifestyle adaptations within this genus.

## Introduction

Fungal and oomycete phytopathogens are a major threat to global food security (Fisher *et al.* 2012). Despite many technological and methodological developments, such as the development of disease-resistant crops, this threat remains a pressing concern for humankind due to emergence of new or adapted species, and a lack of in-depth understanding of disease mechanisms and their genomic basis (Fudal *et al.* 2009; Singh *et al.* 2011; Fisher *et al.* 2012).

Plant-associated fungi and oomycetes can be broadly classified as pathogens, endophytes, or saprotrophs, i.e. they are classified based on their capacity to cause disease symptoms on their host plants. Furthermore, these microbes can be linked to 5 different trophic classes based on their specific feeding behavior (Kabbage *et al.* 2015). Necrotrophs are characterized as pathogens that feed on dead tissue, biotrophs as pathogens that feed on living tissue, and hemibiotrophs are pathogens that go through an initial biotrophic phase before switching to a necrotrophic phase (Oliver and Ipcho 2004). In the same classification model, nonpathogenic species that live within a plant are classified as endophytes, while species that live only on decaying plant material are referred to as saprotrophs. This classification model, which is mainly based on observational data, clearly has limitations, for instance when one species is classified as a necrotroph when interacting with one host but as biotroph when interacting with another (Veloso and Van Kan 2018). Consequently, much research in recent years has focused on establishing the genomic basis underlying differences between species that exhibit different lifestyles. Uncovering these genomic signatures would provide a more reliable method of classification and an increased understanding of host colonization and disease mechanisms, which is of significant importance in developing more effective disease management strategies (O’Connell *et al.* 2012; Ohm *et al.* 2012; Spanu 2012; Möller and Stukenbrock 2017; Plissonneau *et al.* 2017; Haridas *et al.* 2020).

A common feature of various investigations into the genomic basis of pathogenicity is the identification of specific adaptations present in one lifestyle but absent or reduced in the other (Klosterman *et al.* 2011; Gardiner *et al.* 2012; Kim *et al.* 2016). A major current focus is the study of effectors, which are often defined as small secreted proteins that play an important role in establishing the interaction with the host, for instance by degrading the host cell wall or shielding the pathogen from detection by the host immune system (Rovenich *et al.* 2014; Lo Presti *et al.* 2015; Fouché *et al.* 2018). Effectors are often shared by strains and sometimes by species that colonize the same host (Chiapello *et al.* 2015; van Dam *et al.* 2017), and on rare occasions are even passed on to a separate species through horizontal gene transfer (Gardiner *et al.* 2012; van Dam and Rep 2017). The identification of known effectors and other genes that are present only in species of a specific lifestyle can therefore provide useful information when studying the genomic basis of pathogenicity (Gibriel *et al.* 2016). However, as hosts rapidly evolve mechanisms to recognize effectors to re-establish immunity, effectors frequently mutate resulting in rapid effector diversification to avoid detection by the host immune system. Thus, effector repertoires in separate fungal lineages may differ significantly (Rovenich *et al.* 2014; Lo Presti *et al.* 2015).

Carbohydrate active enzymes (CAZymes) play diverse roles in degradation and biosynthesis of carbohydrates such as those found in plant cell walls. For example, plant pathogens can utilize CAZymes to penetrate the host cell wall to establish symbiosis and to liberate carbohydrates from host tissues for growth and reproduction (van den Brink and de Vries 2011; Kubicek *et al.* 2019). Thus, CAZymes can also contribute to virulence, and differences in CAZyme repertoires can mediate microbial lifestyle differences (ten Have *et al.* 2002; King *et al.* 2011; Hane *et al.* 2020). Consequently, CAZymes have been used to propose new lifestyle classification models for oomycete and fungal species (Hane *et al.* 2020). For instance, Hane and colleagues recently proposed 5 new trophic classes based on primary nutrient source preferences as approximated by the presence and abundance of CAZymes directly predicted from genome assemblies (Hane *et al.* 2020): polymertrophs correspond best to necrotrophs, and have received their name due to a preference for polymeric carbohydrates as primary nutrient sources. In contrast, monomertrophs, which correspond best to symbionts and biotrophs, prefer monomeric primary nutrient sources. Mesotrophs are an intermediate group utilizing both monomeric and polymeric nutrient sources, and correspond best to hemibiotrophs. Vasculartrophs are similar to hemibiotrophs, but also include species commonly classified as wilts, anthracnoses, and rots. The saprotrophic class remains a separate group, encompassing species that feed mainly on decaying plant material. These new trophic classes were proposed based on a broad and phylogenetically diverse set of phytopathogenic fungi and oomycetes (Hane *et al.* 2020), including several members of *Dothideomycetes*, a diverse class including many plant-associated fungi as well as fungi adapted to other lifestyles such as marine or soil environments (Haridas *et al.* 2020).

Within the *Dothideomycetes*, members of the genus *Phyllosticta* are particularly well suited to study the genomic basis of lifestyle adaptation and phytopathogenicity. *Phyllosticta* contains at least 50 species that are able to associate with a broad range of plant hosts (Wikee *et al.* 2013b), but species that colonize *Citrus* are of particular interest as they comprise both endophytes and pathogens while being phylogenetically closely related (Wikee *et al.* 2013b; Guarnaccia *et al.* 2019). The most well-known species of this genus is *Phyllosticta citricarpa* which causes Citrus Black Spot, a disease causing significant economic losses worldwide and which therefore has a quarantine status in Europe (Kotzé 2000; European Food Safety Authority 2014; Eustáquio Lanza *et al.* 2018). *Phyllosticta paracitricarpa* is closely related to *P. citricarpa*, bears a strong morphological resemblance and appears to cause similar disease symptoms on *Citrus* (Guarnaccia *et al.* 2017). Other pathogens include *P. citriasiana*, described from several *Citrus* hosts in Asia, *P. citrimaxima*, described from *Citrus maxima* in Thailand, and *Phyllosticta*  *citrichinaensis*, described as a weak pathogen from several *Citrus* hosts in China (Wulandari *et al.* 2009; Wang *et al.* 2012; Wikee *et al.* 2013b). Endophytic species within the genus are *P. capitalensis* with a very broad host range and present on all continents, *P. paracapitalensis*, known from *Citrus* in Europe, and *P. citribraziliensis*, currently known only from *Citrus* in Brazil (Glienke *et al.* 2011; Wikee *et al.* 2013a; Guarnaccia *et al.* 2017).

Genomes were recently published for *P. capitalensis*, *P. citriasiana*, *P. citribraziliensis*, *P. citricarpa*, *P. citrichinaensis*, and *P. paracitricarpa*, with genome sizes ranging between 29 and 32 Mb (Guarnaccia *et al.* 2019). These genomes pave the way for comparative genomic studies aimed to disentangle lifestyle differences within the genus *Phyllosticta* (Guarnaccia *et al.* 2019). Although both mating types (MAT1-1 and MAT1-2) are reported to exist for both endophytic and pathogenic species (Guarnaccia *et al.* 2019; Petters-Vandresen *et al.* 2020), the pathogenic strains for which genomes have been published are all heterothallic and only of the MAT1-2 mating type. In contrast, the sequenced strain of the endophytic *P. citribraziliensis* is heterothallic and of the MAT1-1 mating type, while the other sequenced endophyte*, P. capitalensis*, is homothallic and therefore contains both mating types genes (Guarnaccia *et al.* 2017, 2019; Petters-Vandresen *et al.* 2020). *Phyllosticta citrichinaensis* is also homothallic, but the MAT1-2 idiomorph in *P. citrichinaensis* is not present at the mating-type locus but at another genomic region (Petters-Vandresen *et al.* 2020). This phenomenon sets *P. citrichinaensis* apart from the other species in the genus as the configuration of the mating-type locus is typically very conserved amongst *Phyllosticta* species (Petters-Vandresen *et al.* 2020).

Previous comparative analyses between pathogenic and endophytic *Phyllosticta* spp. have been hampered by the quality of genomes and the availability of only a single endophyte genome (*P. capitalensis*), which was relatively distantly related to the species it was compared to, and consequently genomic adaptations toward these 2 broad lifestyles remained unclear (Wikee *et al.* 2013b; Rodrigues *et al.* 2019; Wang *et al.* 2020). Therefore, a comparison of new and high-quality genomes which includes multiple species of different lifestyles could provide the necessary foundation to finally discovering the genomic underpinning for phytopathology in *Phyllosticta*, which is essential for the development of better disease management strategies.

*Phyllosticta citrichinaensis* was originally described as a weakly aggressive pathogen of several *Citrus* hosts in China as it was isolated from freckles or spots on fruits or leaves of citrus (Wang *et al.* 2012). However, lesions never exhibited typical pycnidia, the presence of this species was never reported to cause any crop or economic losses and Koch’s postulates may not have been established (Wang *et al.* 2012), and thus its lifestyle remains ambiguous. *P. citribraziliensis* is a very close relative of *P. citrichinaensis* and has been described only as an endophyte from Brazil. Therefore, if these 2 species were ascertained to be of 2 different lifestyles, they would be ideal species to study pathogenicity in *Phyllosticta*. As the genome of *P. citrichinaensis* has not been included in earlier comparative work focused on lifestyle differences (Rodrigues *et al.* 2019; Wang *et al.* 2020), a thorough study of its genome and comparison to the genomes of the other species in this genus could provide valuable information on this species’ lifestyle as well as genomic underpinning of disease mechanisms of other species in this genus. Here, we present the first comparative genomics study using multiple complete genomes of 2 endophytic and 3 phytopathogenic *Phyllosticta* species and established genomic differences between species of different lifestyles within this genus. In addition, we use the genomes of 2 separate *P. citrichinaensis* isolates, one of which is newly sequenced, in an attempt to elucidate the lifestyle of the ambiguous *P. citrichinaensis*.

## Materials and methods

### Sequencing, annotation, genome quality, and availability

All non-*Phyllosticta* genomes were previously published (Haridas *et al.* 2020) and are available on MycoCosm (https://mycocosm.jgi.doe.gov/Dothideomycetes; last accessed: 22 March 2022); Grigoriev *et al.*, 2014). The database identifiers (DBIDs) that are given by the Joint Genome Institute (JGI) to identify specific genomes, and which can be used to access the genome’s online portal (https://mycocosm.jgi.doe.gov/DBID, e.g. https://mycocosm.jgi.doe.gov/Aaoar1; last accessed: 22 March 2022) are listed in Supplementary Table 1. Seven of the 8 *Phyllosticta* genomes included in our analyses were also previously published (Guarnaccia *et al.* 2019), and are available on MycoCosm (mycocosm.jgi.doe.gov/Phyllosticta; last accessed: 22 March 2022).

**Table 1.** CATAstrophy predictions for the *Phyllosticta* species analyzed in this study.

	Final Prediction	Trophy scores:				
Species name		Meso	Monomer	Polymer	Sapro	Vascular
*P. capitalensis*	Saprotroph	0.88	0.80	**0.95**	**1.00**	0.00
*P. citribraziliensis*	Saprotroph	0.84	0.82	0.90	**1.00**	0.00
*P. citrichinaensis* (CBS 129764)	Saprotroph	0.82	0.85	0.88	**1.00**	0.00
*P. citrichinaensis* (CBS 130529)	Saprotroph	0.78	0.88	0.84	**1.00**	0.00
*P. citriasiana*	Saprotroph	0.77	0.86	0.84	**1.00**	0.00
*P. citricarpa* (CPC 27913)	Saprotroph	0.82	0.83	0.89	**1.00**	0.00
*P. citricarpa* (CBS 127454)	Saprotroph	0.75	0.88	0.82	**1.00**	0.00
*P. paracitricarpa*	Saprotroph	0.75	0.87	0.82	**1.00**	0.00

Bold values indicate scores of 0.95 or higher.

*Phyllosticta citrichinaensis* liquid cultures (250 ml Malt peptone broth in 500 ml Erlenmeyer flasks) were incubated at 25°C and 180 rpm for 10 to 14 days, after which genomic DNA was isolated using the Qiagen Genomic-tip 100/G kit and the Qiagen Genomic DNA Buffer Set. The genome assembly of *P. citrichinaensis* genome (CBS 129764) was generated by the JGI using the PacBio (>10kb with Blue Pippin Size Selection) long-read sequencing technology. Long-read sequencing data was assembled using Flye 2.3.6 and the genome assembly was annotated using the JGI Annotation pipeline (Grigoriev *et al.* 2014). The genome assembly and annotation are available via the MycoCosm platform (https://mycocosm.jgi.doe.gov/Pcit129764; last accessed: 22 March 2022). Quality assessments were performed using BUSCO 4.1.4 (Manni *et al.* 2021) and QUAST 5.0.2 (Gurevich *et al.* 2013) using default parameters. One-to-one whole-genome comparisons were performed using PROmer (default settings), which is part of the MUMMer 3.25 conda package (Marçais *et al.* 2018) and plotted with mummerplot (also part of MUMMer) using the –filter and –fat parameters. We used OrthoFinder 2.2.6 (Emms and Kelly 2019) to identify ortholog groups (OGs) across all 116 fungal genome annotations (Supplementary Table 2a). Ortholog groups unique to species of specific lifestyles were identified using UpSetR 1.4.0 (Supplementary Table 2b, Conway *et al.* 2017).

**Table 2.** CAZyme families with gene abundance differences between endophytes and pathogens.

Species name	Traditional lifestyle	CBM67	AA1_3	CBM18	GH3	PL22	AA3
*Phyllosticta capitalensis*	E	0	10	10	15	1	2
*Phyllosticta citribraziliensis*	E	0	10	9	15	1	2
*Phyllosticta citrichinaensis* (CBS 129764)	?	0	10	9	16	1	1
*Phyllosticta citrichinaensis* (CBS 130529)	?	0	10	9	15	1	1
*Phyllosticta citriasiana*	P	1	9	7	13	0	1
*Phyllosticta citricarpa* (CPC 27913)	P	1	9	6	14	0	1
*Phyllosticta citricarpa* (CBS 127454)	P	1	9	7	14	0	1
*Phyllosticta paracitricarpa*	P	1	9	7	14	0	1

Columns with a darker fill color indicate the family which is more abundant in pathogens, while the lighter color indicates those which are more abundant in endophytes. E = Endophyte, P = Pathogens, ? = Lifestyle ambiguous.

### Secreted proteins and effectors

Predector 1.1.0 was used to predict secreted proteins and effectors in the predicted proteomes of all 116 fungal genomes. We used predicted secreted proteins with a manual secreted core > 4, and effectors with an effector score > 1 or > 2 in further analysis. We visualized the distribution of OGs of which 50% or more of the genes were predicted to be a secreted protein or an effector, by generating a clustered heatmap in R using the ComplexHeatmap 2.6.2 package (Gu *et al.* 2016).

### Carbon utilization and CATAstrophy

Carbon growth studies were performed as described previously (Buijs *et al.* 2021). In short, 1-mm-diameter plugs from 2-week-old colony edges of *Phyllosticta* species were inoculated on 35 different carbon sources and incubated at 25°C until the largest colony reached the edge of a 35-mm-diameter plate. As different *Phyllosticta* species demonstrate different growth speeds, this moment fell on different days after inoculation (between 5 and 10 days). When the largest colony of a species reached the edge of a plate, colony diameters were measured on all sources and images were taken using a standard camera setup. This approach was chosen to be able to compare species with different growth speeds. All growth studies were performed in duplicate. Measurements were averaged and used to generate a clustered heatmap using the ComplexHeatmap 2.6.2 package (Gu *et al.* 2016) in R (R Core Team, 2021).

We used CATAstrophy to predict lifestyles from CAZyme repertoires (Hane *et al.* 2020). To this end, we first used hmmpress 3.3.2 to generate a local HMMER database of dbCAN 8 (Zhang *et al.* 2018). We then queried all 116 predicted proteomes with the local dbCAN HMMs database using hmmscan 3.3.2 with the –domtblout parameter to create a domain table for each proteome. CATAstrophy 0.0.3 was then ran on all 116 domain tables using parameters –p, –c, –model v8 and –format hmmer_domtab. The heatmap was created by identifying all OGs (as created previously using OrthoFinder) that contained a CAZyme, counting the number of genes in each OG for each species, and then generating a heatmap using the ComplexHeatmap 2.6.2 package in R. Empty columns, e.g. CAZyme families for which no genes present in these species, were filtered out of the heatmap for visualization purposes, but are present in the original data (Supplementary Table 5).

## Results

### *Phyllosticta* genome assemblies are of high quality

**Fig. 1.**
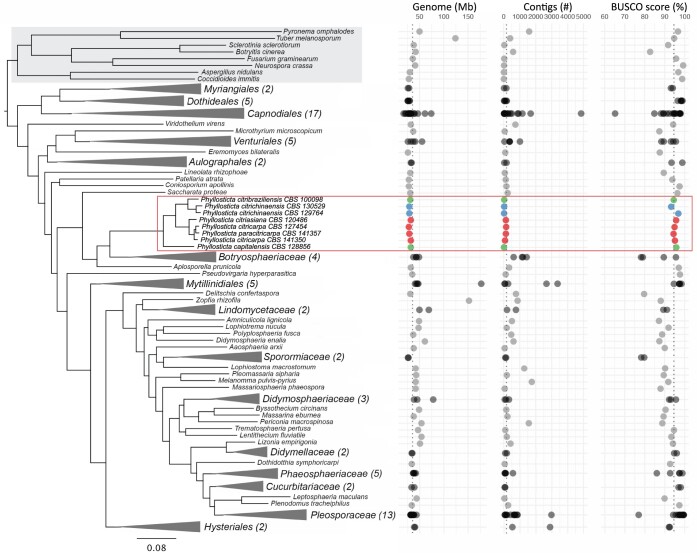
*Phyllosticta* genomes are of high quality. The phylogenetic relationship of more than 100 fungal species (116 genomes) is shown. The phylogenetic tree was generated using OrthoFinder, and sub-trees were collapsed manually to enhance readability. Gray box indicates species outside the *Dothideomycetes*, red rectangle indicates the genus *Phyllosticta*. Red orbs = *Phyllosticta* pathogens, green orbs = *Phyllosticta* endophytes, blue orbs = *P. citrichinaensis.*

Lifestyle differences are often driven by genomic adaptations (Ohm *et al.* 2012; Kabbage *et al.* 2015; Haridas *et al.* 2020), and we hypothesize that this also applies to *Phyllosticta* species with different lifestyles. Taxonomically, *Phyllosticta* belongs to *Dothideomycetes*, a fungal class with extensive genomic resources (Wikee *et al.* 2013b; Haridas *et al.* 2020). To enable studies in lifestyle differences in *Phyllosticta*, we made use of 8 *Phyllosticta* genome sequences, 7 of which were assembled and published previously (Guarnaccia *et al.* 2017, 2019). Here, we performed genome sequencing of *P. citrichinaensis* (CBS 129764), which is the second genome of this species to be sequenced, thereby enabling us to also evaluate intra-species variation. We included genome assemblies of 2 different *P. citrichinaensis* strains, as well as the genome assemblies of the endophyte *P. citribraziliensis*, the closest relative of *P. citrichinaensis*, those of the 2 pathogenic species *P. citricarpa* and *P. paracitricarpa*, which are very closely related (Fig. 1a, also see Guarnaccia *et al.* 2019), of the pathogenic species *P. citriasiana*, and of the endophyte *P. capitalensis*, which is phylogenetically the least related to the other *Phyllosticta* species (Fig. 1a). As these genome assemblies include multiple species of both lifestyles (pathogens and endophytes), comparative genomics may reveal the genomic underpinning for lifestyle adaptations within this genus, and ultimately aid in determining the lifestyle of *P. citrichinaensis*.

To determine genome assembly size, fragmentation, and annotation completeness of the *Phyllosticta* genomes, we used QUAST (Gurevich *et al.* 2013) and BUSCO (Manni *et al.* 2021), and compared the results to 100 previously published *Dothideomycete* genomes as well as 8 genomes of fungal species outside of *Dothideomycetes* (Fig. 1a; Supplementary Table 1, Haridas *et al.* 2020). Compared to the other fungal species, *Phyllosticta* genome assemblies are of a slightly smaller size (29–32 Mb) as opposed to an average of 40 Mb in other *Dothideomycetes* (Fig. 1a). The *Phyllosticta* genome assemblies have a low number of contigs, namely 14–152 contigs compared with on average 471 contigs, and BUSCO scores between 93.3% and 95.8%, suggesting that the *Phyllosticta* genome assemblies are *en par* or above-average quality in terms of genome contiguity and completeness compared to the other *Dothideomycetes* genomes, which should facilitate further comparative analyses.

### *Phyllosticta* differ in gene number and functional annotation in a lifestyle dependent manner

**Fig. 2.**
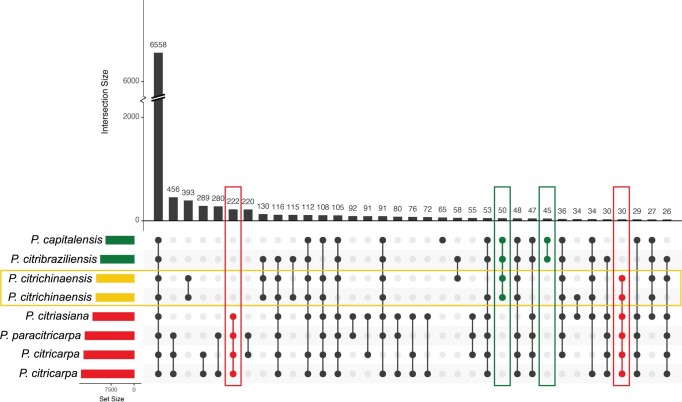
*Phyllosticta citrichinaensis* shares more unique OGs with endophytes than with pathogens, but pathogens share more OGs with each other. Yellow rectangles = *P. citrichinaensis*, red rectangles = pathogens, green rectangles = endophytes. Figure generated using UpSetR. Only the largest 35 groups are shown.

Species of similar lifestyles often share (groups of) genes (Lo Presti *et al.* 2015; Kim *et al.* 2016). Consequently, we hypothesized that the presence or absence of specific genes may provide information about the (predominant) lifestyle of *P. citrichinaensis*. To be able to compare gene content over different species and strains, we used OrthoFinder (Emms and Kelly 2019) to identify ortholog groups (OGs) across all 116 predicted proteomes. Orthofinder identified 35,379 OGs containing 88.1% of all genes (Supplementary Table 2a). The 11.9% of genes that were not assigned to any OG likely constitute species-specific genes, which is to be expected given the taxonomically diverse set of fungal species considered in our study (Fig. 1a). In total, 1,794 OGs (5.1%) contained genes from all species, representing a fungal core genome. Of all OGs, 32.2% (11,352) contained at least1 gene from a *Phyllosticta* species, and of those, 57.8% (6,558, Fig. 2) contained genes from all *Phyllosticta* species and 33.2% (3,764) were unique to *Phyllosticta* (i.e. they only contained *Phyllosticta* genes). The latter percentage is quite high because of the close taxonomic relation of some of the genomes: a rather large fraction of the *Phyllosticta* unique OGs contain 2 or 3 genes (2,734, 72.6%), as these often contain 1 gene from each of the 2 *P. citricarpa* genomes and one more gene from *P. paracitricarpa*. Genes that are unique to a species, i.e. sequences that are sufficiently different from other sequences, do not form a separate OG on their own and consequently are not considered in these statistics. Since the *P. citricarpa* and *P. paracitricarpa* genomes are so closely related, many of their “unique genes” are assigned to an OG, which causes the fraction of *Phyllosticta* unique OGs to be quite large.

To discover lifestyle-associated genes in *Phyllosticta*, we compared OG content across the 8 *Phyllosticta* species and sought to identify differences between species of different lifestyles. The number of OGs that is shared by *Phyllosticta* species of a specific lifestyle (45–222, Fig. 2 and Supplementary Table 2b) is much smaller than the total number of OGs that all species in this genus share (6,558). The OGs shared by *Phyllosticta* spp. of a specific lifestyle might also contain genes from species outside of this genus. *Phyllosticta* pathogens shared 222 OGs that are not present in *Phyllosticta* endophytes, a much higher number than the total of 45 OGs that are shared by endophytes and not present in pathogens, which is likely due to the larger phylogenetic distance between the 2 endophytes compared with the pathogenic *Phyllosticta* species (Fig. 1a). The larger phylogenetic distance of *P. capitalensis* is also apparent through the fact that it has a group of 65 OGs that are not shared with any of the other *Phyllosticta* species. This is a rather large number when compared to the other *Phyllosticta* species, which all had less than 25 OGs that were not shared with the other *Phyllosticta* species. In addition to OGs that are unique to species of a specific lifestyle, OGs that are present in all species but differ in their abundance in species that share a lifestyle (e.g. there are more genes in species of 1 lifestyle compared to the others) may provide information on how species adapt to their lifestyles. We thus identified OGs for which the average number of genes in species of 1 lifestyle was higher than the average number of genes for species of the other lifestyle; we did not consider OGs that contained large outliers, i.e. one species having a much larger number of genes as compared to the other species. This resulted in a total of 87 OGs: 73 OGs that had more genes in endophytes and 14 OGs that had more genes in pathogens (Supplementary Table 2c), suggesting that achieving the endophytic lifestyle requires additional genes.

As functions of genes in OGs that are unique to, or enriched in, species of a certain lifestyle may underly lifestyles adaptations, and this may help to uncover which lifestyle *P. citrichinaensis* has, we looked into the annotations of the lifestyle-related OGs. In addition to the annotations of *Phyllosticta* genes, we also included the annotations of 50 out of the 108 species outside the genus *Phyllosticta* for which annotation data was available on the JGI database. The number of OGs in which at least one gene was functionally annotated (other than as hypothetical or expressed protein) varied widely between different lifestyle-related groups: while nearly 50% of the OGs that had more genes in endophytes received an annotation, less than 3% of the pathogen-only group did (Supplementary Table 3, a–f). We further divided the individual functional annotations into KOG-classes (from the EuKaryotic Orthologous Groups tool, https://mycocosm.jgi.doe.gov/help/kogbrowser.jsf; last accessed: 22 March 2022), which provide a high-level classification system to group genes with comparable activities. Of all KOG-classes, the class “Secondary metabolites biosynthesis, transport, and catabolism” was most often found to be associated with lifestyle-related OGs: only 2 of 6 lifestyle-related groups did not contain genes in this class, suggesting this group of genes may be useful to distinguish species of different lifestyles (Supplementary Table 3i).

To study secondary metabolite biosynthesis genes in more detail we used antiSMASH (Blin *et al.* 2019) to identify biosynthetic gene clusters (BGCs) in the 8 *Phyllosticta* genomes. The total number of predicted BGCs varied from 20 in *P. paracitricarpa* to 24 in *P. citricarpa*, with no apparent differences in numbers between species of different lifestyles. Interestingly, one of the terpene clusters was predicted as a squalestatin in all pathogenic species, while it received no functional prediction in the endophytic species or in *P. citrichinaensis*, suggesting there is a difference in this cluster between species of different lifestyles. Squalestatins are predicted to be inhibitors of squalene synthase, which produces squalene, a sterol biosynthetic intermediate that is reported to play a role in mediating interactions between fungi and their plant hosts (Lindo *et al.* 2020). Therefore, further characterization of this BGC and others in *Phyllosticta* will be worthwhile for future studies into pathogenicity of *Phyllosticta* species.

Ortholog groups with more genes in *Phyllosticta* endophytes were more often functionally annotated, suggesting that these are generally better characterized and likely evolutionary conserved. We did not find any particular KOG-class to be annotated in higher abundance in endophytes, but nonetheless found a few interesting annotated OGs, such as 6 OGs that were annotated to belong to the “carbohydrate transport and metabolism” class including a CAZyme family (GH55) gene and several transporters, suggesting a role for carbohydrate transport in lifestyle (Supplementary Table 3, a–f, i). One endophyte-only OG contained the MAT1-1 gene, which was the result of all sequenced pathogenic strains having MAT1-2 mating types (Petters-Vandresen *et al.* 2020). In addition, although the *P. citrichinaensis* MAT1-1 gene is not present in this OG, we did find the MAT1-1 gene in the *P. citrichinaensis* genome assembly and found it to be highly similar to that of *P. citribraziliensis*, its closest relative.

The pathogen-only group was poorly functionally annotated; out of 222 OGs, only 5 received a functional annotation. The fact that such a large fraction of OGs could not be assigned a functional annotation is of interest as some of the nonannotated OGs in the pathogen-only group could contain putative effectors or genes that are otherwise involved in virulence, as effectors often remain unannotated in standard annotation pipelines (Sperschneider *et al.* 2015). In addition, 2 OGs received functional annotations that have previously been implied to be virulence factors and could therefore be interesting targets for future functional studies; a pectin lyase fold (Yang *et al.* 2018), and a cytochrome p450 (Siewers *et al.* 2005; Shin *et al.* 2017).

### *Phyllosticta citrichinaensis* shares more lifestyle-specific OGs with endophytes, but follows an intermediate pattern in other lifestyle-associated OGs

The lifestyle of *P. citrichinaensis* is currently ambiguous (Wang *et al.* 2012), but the number of OGs that it shares with species of either lifestyle may provide clarity not only about the lifestyle of *P. citrichinaensis* itself but also about the differences between species of different lifestyles within this genus. The 2 *P. citrichinaensis* strains share more lifestyle specific OGs with endophytes (50) than they do with pathogens (30). In addition, the number of OGs that is shared by only the 2 endophytes and *P. citrichinaensis* is larger than the number of endophyte-only OGs that are not shared with *P. citrichinaensis* (Fig. 2, green rectangles), suggesting that *P. citrichinaensis* indeed compared well to endophytic species. However, for 30 out of 87 OGs that contained more genes in either pathogenic or endophytic species, the *P. citrichinaensis* gene numbers corresponded best to the endophytic numbers, while in 32 OGs, they corresponded to the pathogenic numbers. In 25 OGs, the number of genes of *P. citrichinaensis* corresponded to neither lifestyle (Supplementary Table 2c). As opposed to the numbers of OGs specific to species of one lifestyle, where *P. citrichinaensis* shared more with endophytes (Fig. 2), the numbers of *P. citrichinaensis* genes in OGs that contained more genes in either pathogenic or endophytic species thus indicate that it compares equally well to species of either lifestyle. Thus, these data suggest that presence/absence and/or gene abundance differences are not sufficient to provide insights into the lifestyle of *P. citrichinaensis.*

While we did not observe clear patterns in the types of functions that *P. citrichinaensis* shares with either endophytes or pathogens, we nevertheless could observe some interesting functional patterns in *P. citrichinaensis* (Supplementary Table 3, g and h). For instance, 2 groups were annotated as heat shock proteins: an Hsp40 (DNAJC17) that had more genes in pathogens (3 in pathogens vs 1–2 in endophytes) as well as an Hsp70 that had more genes in endophytes (4–5 in pathogens vs 6–7 in endophytes). *Phyllosticta citrichinaensis* contains fewer genes in both groups, suggesting it may respond differently to stress. Furthermore, 1 group that had more genes in pathogens as well as in *P. citrichinaensis* (1 in endophytes vs 2–3 in pathogens) contains genes annotated as peroxiredoxin-1 or peroxiredoxin-6. Peroxiredoxins are necessary for full virulence in several fungal pathogens such as *Magnaporthe oryzae* and *Aspergillus fumigatus* as they offer an antioxidant defense against reactive oxygen species produced by the host as part of host defense responses (Mir *et al.* 2015; Rocha *et al.* 2018). It is thus possible that these additional genes in *Phyllosticta* pathogens and in *P. citrichinaensis* contribute to their virulence.

### There is little difference between *Phyllosticta* endophytes and pathogens in the numbers of putative secreted proteins and putative effectors

**Fig. 3.**
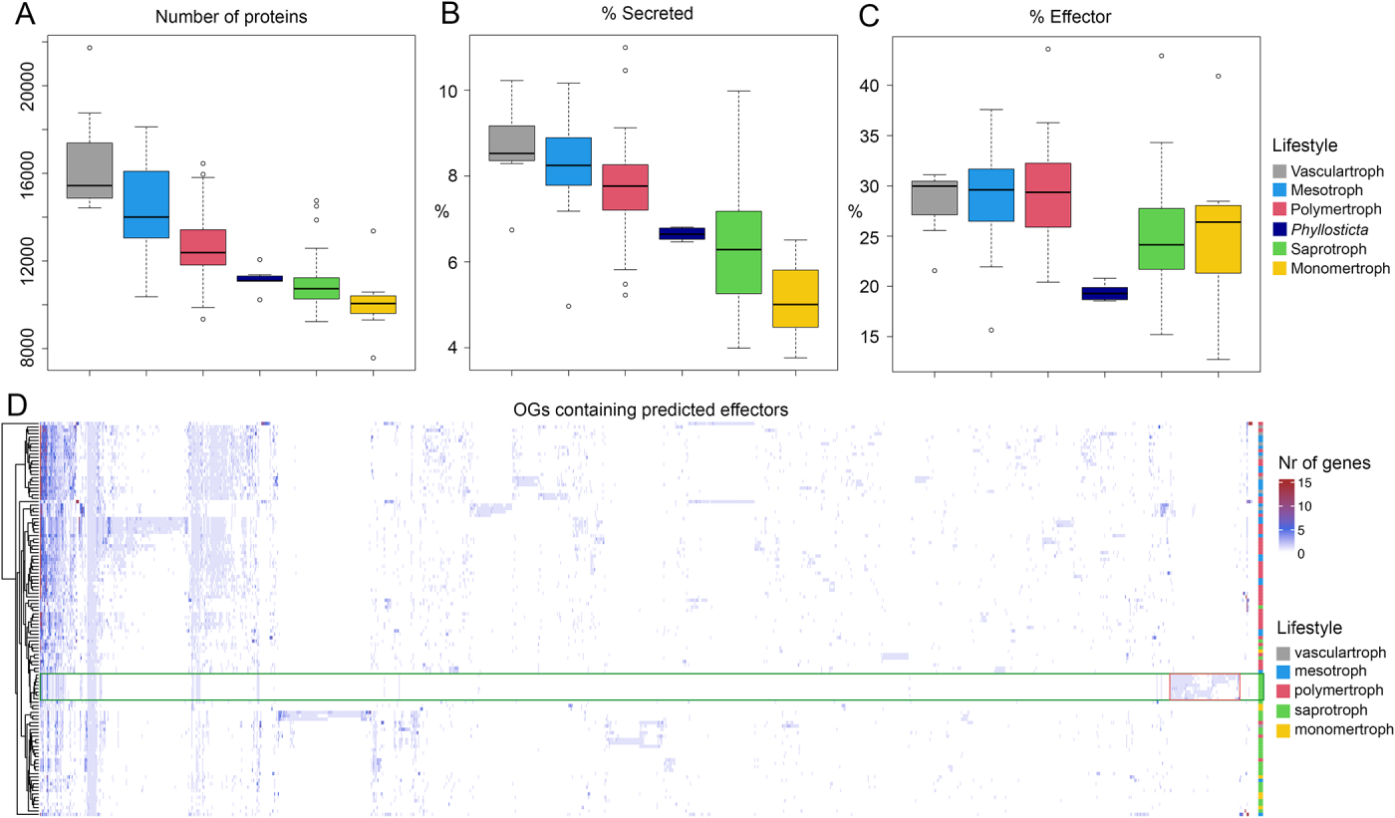
Species cluster independent of their taxonomic relationship based on presence effector proteins. a) The total number of proteins, b) the percentage of proteins that is predicted to be secreted, and c) the percentage of secreted proteins that is predicted to be an effector, in species of different lifestyles (as predicted by CATAstrophy, see Fig. 5). d) Clustered heatmap of the number of genes in OGs that were predicted to contain effectors. Lifestyles as predicted by CATAstrophy (see Fig. 5). *Phyllosticta* species are highlighted with green rectangle, *Phyllosticta*-unique OGs are highlighted in red rectangle.

Secreted proteins, including effectors, play an important role in lifestyle and virulence (de Wit *et al.* 2009; Lo Presti *et al.* 2015; Plissonneau *et al.* 2017). Based on the comparison of OGs in species of different lifestyles, we concluded that pathogenic species contain a large number of unannotated genes in pathogen-specific OGs (217 OGs). We hypothesized that some of these genes may be effectors or other secreted proteins, and that the presence of putative secreted proteins and effectors in the genomes of species may be an indicator for lifestyle differences. We therefore assessed the presence of OGs that contain 50% or more putative secreted or effector proteins in all 116 species used in this study. We used Predector (Jones *et al.* 2021) with a manual secretion score >4 to predict secreted proteins and an effector score >1 to predict effectors. A total of 3,537 OGs (10% of 35,379 OGs) consisted of at least 50% secreted proteins (Fig. 3b, Supplementary Fig. 1, Supplementary Table 4a) and of these, 1,315 OGs consisted of at least 50% proteins with an effector score > 1 (Fig. 3, c and d, Supplementary Table 4b). Increasing the effector score threshold to >2 resulted in a large decrease in predicted effectors. Notably, only 34 OGs containing putatively secreted genes were present in all 116 species, and only 1 OG containing putative effectors contained genes from all 116 species, corroborating that secreted proteins and effectors are typically not shared between different species and especially effectors are rather species and/or strain specific (Stergiopoulos *et al.* 2012).

*Phyllosticta* genes were present in a total of 674 putatively secreted OGs, with 322 of those containing genes from all *Phyllosticta* species included in this study. With an average of 642 genes per species, *Phyllosticta* species contain less genes in OGs encoding secreted proteins compared with other *Dothideomycetes* (an average of 848 genes, Supplementary Table 4). In addition, *Phyllosticta* species contained on average 152 putatively secreted genes that were not in an OG or that were the only protein predicted to be secreted in an OG (singletons), which is slightly more than we observed for the overall average of 144 singleton secreted genes (Supplementary Table 4). When assessing the total number of predicted secreted genes (both in OGs and singletons), *Phyllosticta* endophytes have more putative secreted proteins (average of 814) as compared to *Phyllosticta* pathogens (average of 795). However, when considering these as a percentage of the total predicted proteome size, this difference becomes negligible, with endophytes having a slightly smaller percentage of proteins that is secreted (7.03%) as compared to pathogens (7.10%, Supplementary Table 4c). We found 288 putatively secreted OGs to be unique to *Phyllosticta*, none of which contained functionally annotated genes, as is often the case for putative effectors (Lo Presti *et al.* 2015).

*Phyllosticta* species contain less genes in OGs encoding effectors with an average of 116 genes compared to the overall average of 177 genes. In addition, *Phyllosticta* species contained on average 27 putative effector genes that were not in an OG or that were the only effector in an OG (singletons), which is lower than the average of 46 singleton effector genes for the other *Dothideomycetes*. Comparable to secreted proteins, when assessing the total number of predicted effector genes (both in OGs and singletons), *Phyllosticta* endophytes appear to have slightly more putative effector genes (average of 149) as compared to *Phyllosticta* pathogens (average of 144), but when taken as percentage of the total number of predicted proteomes per species, this difference is negligible (1.28% vs 1.29%) (Fig. 3, c and d, Supplementary Table 4d). Effector genes are often hypothesized to be species and/or strain specific (Lo Presti *et al.* 2015; Sperschneider *et al.* 2015). We identified in total 63 OGs containing effectors that are unique to *Phyllosticta*, 3 of which were present in all pathogens but not in endophytes, 2 were present in all endophytes but not in pathogens, and 1 had higher gene numbers in endophytes (Supplementary Table 4e). None of these unique effector genes were functionally annotated, suggesting that these have yet undescribed functions.

The occurrence of *P. citrichinaensis* effector genes in lifestyle-associated OGs could provide further evidence for its lifestyle. One of the 3 effector OGs that only occurred in pathogens contained a gene from one of the *P. citrichinaensis* strains. In contrast, both endophyte-only effector OGs contained genes from *P. citrichinaensis*, in one case only from one strain, and in the other case from both strains. In the effector OG that had more genes in endophytes, *P. citrichinaensis* followed an intermediate pattern: one strain contained the same number of genes as pathogens, while the other contained the same number as endophytes. These data thus suggest that *P. citrichinaensis* follows an intermediate lifestyle.

### *Phyllosticta citrichinaensis* clusters with pathogens based on carbon growth data

**Fig. 4.**
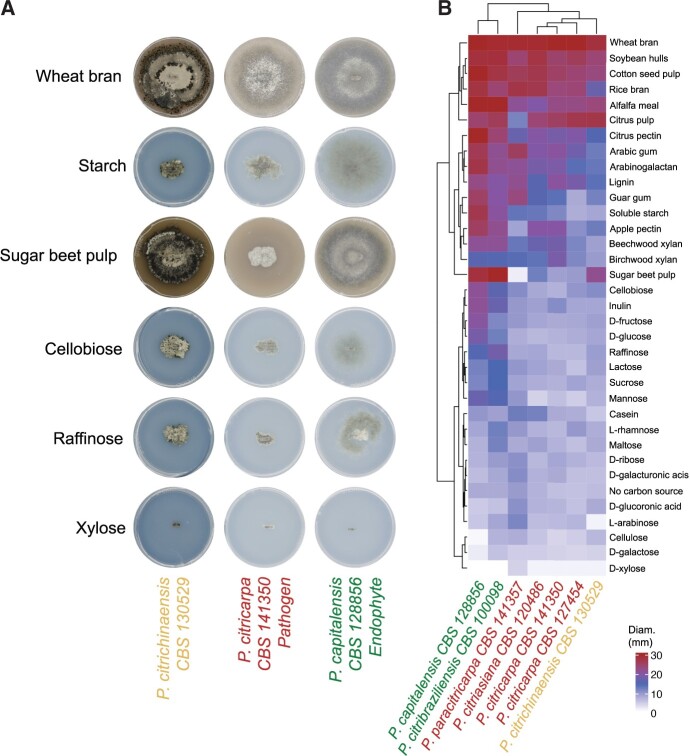
*Phyllosticta citrichinaensis* clusters with pathogens based on growth on 35 carbon sources, but behaves like an endophyte in the presence of sugar beet pulp. a) Images of *Phyllosticta* species growing on a selection of different carbon sources. b) Clustering of *Phyllosticta* species based on their growth on different carbon sources. All species were grown on 35 different carbon sources, colony diameters were measured, and images takes on all sources when the biggest colony of a species reached the edge of its plate. All species grew fastest on wheat bran.

CAZymes enable fungi to utilize different carbon sources (van den Brink and de Vries 2011; Lombard *et al.* 2014), and are thought to be involved in fungal pathogenicity (ten Have *et al.* 2002; King *et al.* 2011; Kubicek *et al.* 2014; Hane *et al.* 2020). We have previously shown that carbon utilization capabilities differ between *Phyllosticta* spp. and uncovered a clear distinction in the ability of pathogens and endophytes to grow in the presence of sugar beet pulp; while the growth of endophytes was unchanged, pathogens were strongly inhibited (Buijs *et al.* 2021). To assess if *P. citrichinaensis* displays similar growth behavior to pathogens or endophytes, we grew *P. citrichinaensis* on 35 different carbon sources including sugar beet pulp (Fig. 4). Interestingly, growth of *P. citrichinaensis* is not inhibited by the presence of sugar beet pulp (Fig. 4a), suggesting that *P. citrichinaensis* behaves comparable to endophytic *Phyllosticta* spp. To further substantiate this observation, we performed hierarchical clustering of 7 *Phyllosticta* strains based on their growth on all 35 carbon sources. Unexpectedly, *P. citrichinaensis* clustered together with the pathogenic species rather than with the endophytes (Fig. 4b). Thus, although *P. citrichinaensis* is not inhibited by sugar beet pulp, it generally displays carbon utilization capabilities comparable with pathogens.

### Genomes of *Dothideomycetes* can be clearly distinguished based on CAZyme content, but this does not correlate well with lifestyles described in literature

**Fig. 5.**
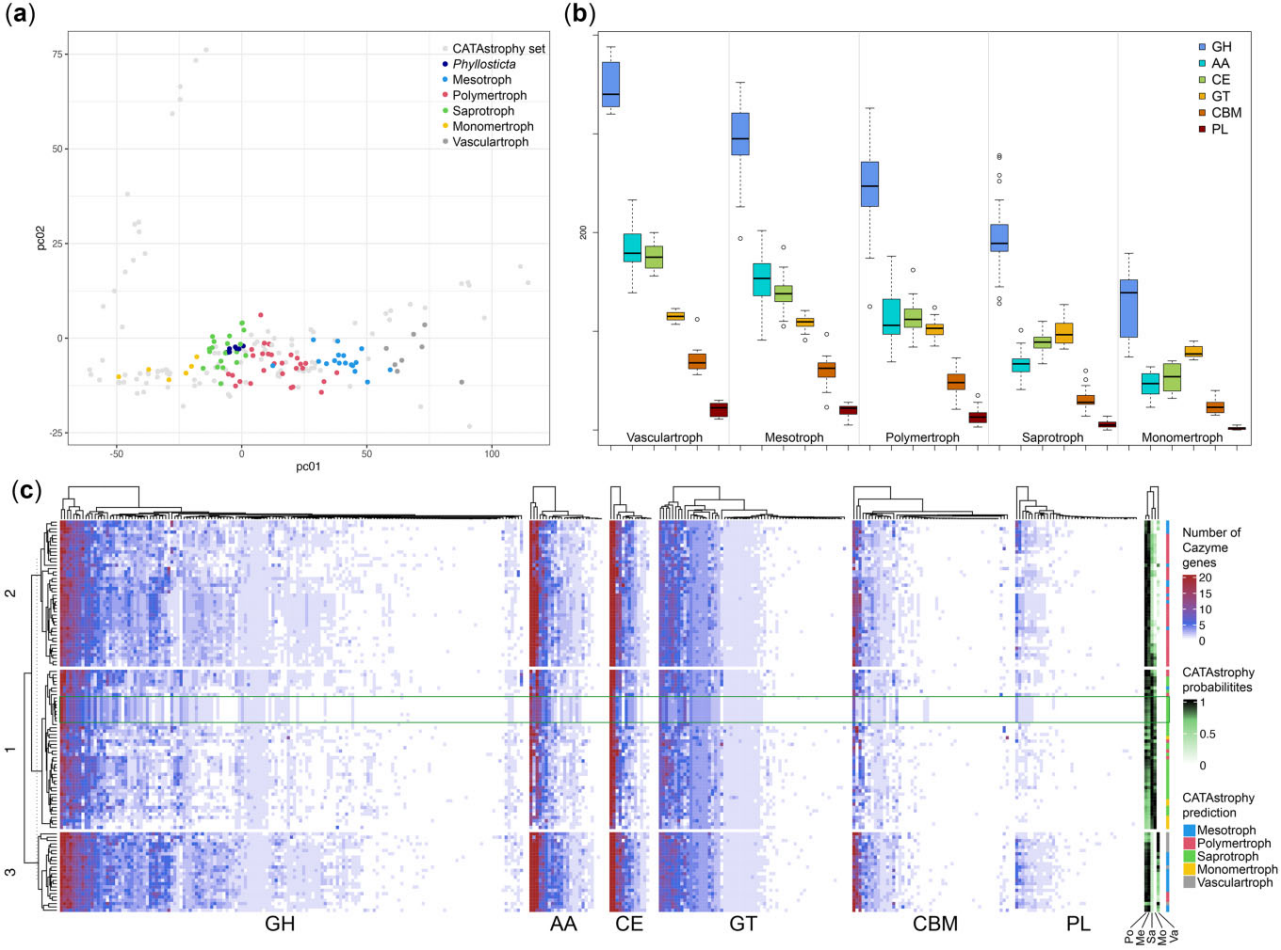
Separation of species into different trophy classes based on presence of CAZyme genes in their genomes. a) PCA plot of PC1 vs PC2. PC1 separates species of different trophy classes, PC2 separates species on phylogeny. The CATAstrophy dataset published by Hane *et al.* (2020) is shown in light gray for comparison. b) Number of CAZyme genes in each CAZy family per CATAstrophy class. c) Clustered heatmap of the number of CAZyme-gene-containing OGs per species. GH, glycosyl hydrolase; AA, auxiliary activity; CE, carbohydrate esterase; GT, glycosyl transferase; CBM, carbohydrate binding module; PL, pectin lyase; Po, polymertroph; Me, mesotroph; Sa, saprotroph; Mo, monomertroph; Va, vasculartroph.

The genetic basis for the ability to utilize different carbon sources is often caused by differences in CAZymes repertoires (van den Brink and de Vries 2011; Lombard *et al.* 2014). Interestingly, the abundance and diversity of CAZymes encoded in a genome is also related to lifestyle and consequently enables to predict the tropic classification of a species (Lo Presti *et al.* 2015; Hane *et al.* 2020). Our dataset of 116 genomes included 27 species whose trophy classification was previously predicted by Hane *et al.* using CATAstrophy. We used CATAstrophy to annotate CAZyme genes for the other 89 predicted proteomes and to perform a principal component analysis (PCA) to distinguish species with different trophic classes based on their CAZyme repertoire. CATAstrophy clearly separated species of different trophic classes based on the first principal component (PC1) (Fig. 5a and Supplementary Table 5). As the second principal component (PC2) mainly separates oomycetes from fungi (Hane *et al.* 2020), we did not observe much separation based on PC2 as the here analyzed genomes did not include any oomycetes. For these 116 species, the different trophic classes differ considerably in the numbers of genes per CAZyme family (Fig. 5b). For example, GH families differ clearly between trophy class, while almost no difference can be observed in the PL family. The CATAstrophy gene predictions were used to identify CAZyme-containing OGs, for which we then obtained the number of genes present in each species to generate a clustered heatmap (Fig. 5c). We observed 3 distinct clusters that differ in their CAZyme repertoires, which typically correlate well with the CATAstrophy trophy predictions (Fig. 5c and Supplementary Table 5). The CATAstrophy trophy predictions also correlate well with the numbers of secreted proteins (predictor threshold >4) and effectors (predictor threshold >1 and >2), although we did not observe such a strict separation into 3 clusters as was observed for CAZyme genes (Figs. 3 and 5c, Supplementary Fig. 1).

All *Phyllosticta* species were predicted to be saprotrophs by CATAstrophy (Table 1, Fig. 5c) and clustered according to their phylogenetic relationship, suggesting that they are generally very similar in terms of CAZymes (Supplementary Table 5). Consequently, *P. citrichinaensis* clustered most closely to *P. citribraziliensis*, an endophyte, as this is its closest relative (Fig. 1a). We nevertheless found 6 CAZyme families that show consistent differences in gene number between pathogens and endophytes: AA1_3, AA3, CBM18, CBM67, GH3, and PL22 (Table 2). In all cases except AA3, *P. citrichinaensis* follows the endophytic pattern. Together, these results indicate that in terms of the presence of CAZyme genes, the *P. citrichinaensis* genomes compare best to those of endophytes.

## Discussion

Lifestyle adaptations are thought to be driven by differences in gene content, and especially CAZymes are assumed to be crucial (ten Have *et al.* 2002; King *et al.* 2011; Hane *et al.* 2020). Here, we aimed to elucidate the genomic differences between endophytes and pathogens within the *Phyllosticta* genus occurring on *Citrus*, and aimed to determine the lifestyle of *P. citrichinaensis*. Based on the results, we uncovered several differences between species with different lifestyles. For instance, endophytes more frequently contain higher numbers of genes in OGs, and these genes are more often annotated than in pathogenic species. In addition, pathogenic species share more un-annotated lifestyle-specific OGs as compared to endophytic species. Furthermore, we show that species cluster independently of phylogeny based on the CAZyme content of their genomes, and this clustering correlated well with trophic lifestyle prediction by CATAstrophy. The ambiguous species *P. citrichinaensis* showed characteristics that matched with endophytes in some cases, with pathogens in other cases, and sometimes it did not match with either lifestyle, suggesting it may exhibit an intermediate lifestyle not accounted for in the current definitions.

We previously observed that only 4 CAZyme families showed a consistent difference between endophytic and pathogenic species (Buijs *et al.* 2021), one of which contained AA1_3/CBM18 (7 in pathogens vs 8 in endophytes), which were mixed in one orthologue group, and another one contained family CBM18 only. In this study, we found in total 6 CAZyme families with a consistent difference between endophytes and pathogens. These included 2 separate OGs that contained the AA1_3 and CBM18 CAZyme families, which both consistently contained more genes in endophytes compared to pathogens. CATAstrophy predicts on average about 90 CAZyme genes extra compared to our previous results (Buijs *et al.* 2021). The biggest difference can be found in the CE (carbohydrate esterase) family, where between 83 and 89 genes are predicted instead of 15–17. Most of these are in the CE10 family (47–53). The CE10 family is no longer listed as carbohydrate active enzyme by the cazy.org database (used by JGI) because most of the members of this family act on noncarbohydrate substances (Lombard *et al.* 2014). If we manually remove these, CATAstrophy still predicts 20 extra genes in the CE family. In addition, there are some 15–20 extra genes predicted in the AA family, 5 to 10 more in the GH family, and approximately 5 more in the GT family. Numbers in the PL family are practically identical. In the CBM family, CATAstrophy predicts about 5 to 10 genes less. In the JGI annotation pipeline, many of the genes in the CBM family contained multiple domains and were therefore counted multiple times, which might have led to an overestimation. However, for most families it seems that CATAstrophy predicts more genes compared to JGI. As most of the CAZyme genes predicted by JGI are based on some experimental validation (cazy.org), it would be wise to experimentally validate the CAZyme genes that were predicted by CATAstrophy and not by JGI.

Genes related to secondary metabolite biosynthesis but not the number of BGCs as predicted by antiSMASH differ between species. It is important to note here that BGC prediction by antiSMASH is based primarily on the presence of a gene to produce the metabolite ‘backbone’, such as a polyketide synthase (PKS) (Blin *et al.* 2019). Once such a gene is found, antiSMASH takes an area in the genome of up to 20 kb (depending on the type of backbone gene) on either side of the gene and checks for the presence of tailoring genes, which are then all automatically included in the BGC. This means that tailoring genes that are altered or inactive are still included in the predicted BGC, and that alterations in genes may not result in an altered BGC prediction. In contrast, although the cluster may look very similar, alterations in tailoring genes may lead to the production of a very different compound: a good example is the synthesis of the toxins dothistromin, aflatoxin, and sterigmatocystin, which are all synthesized in a very similar manner, with only the very last tailoring steps being different (Schwelm and Bradshaw 2010; for a review see Hüttel and Müller 2021). We therefore conclude that although antiSMASH did not detect differences in BGC numbers, alterations in biosynthetic genes may be responsible for the differences between species of different lifestyles in the genus *Phyllosticta*, and this will be an interesting subject for future studies.

CATAstrophy was able to clearly separate species based on the number of CAZyme genes present in the genomes and was able to separate species into different trophy predictions. Apart from closely related species that cluster together, a phylogenetic pattern cannot be observed in the clustering of the heatmap or in the trophy predictions, which suggests that there is a strong signal that links genome content to lifestyle. CATAstrophy also allows for trophy-overlap for species that are bordering 2 trophies, such as for *Sclerotinia sclerotiorum*, which received very high scores for both the polymertroph and the saprotroph class (Supplementary Table 5). This is consistent with literature, as *S. sclerotiorum* has been described to exhibit a necrotrophic phase that is followed by a saprotrophic phase (Hegedus and Rimmer 2005). *Sclerotinia sclerotiorum* is a much-researched model organism, and the descriptions in literature of its lifestyle are therefore well-developed. However, for many fungal species, this is not the case, and circumscriptions in literature are often limited or conflicting. Indeed, we see that the trophy predictions by CATAstrophy do not always correlate well with lifestyles described (or supposed) in literature: each trophy class includes species that are described as pathogens, endophytes, symbionts, or saprotrophs, or which have been described to exhibit multiple of these lifestyles. An underlying cause for this fact is that lifestyles that are described in literature may be inaccurate as the border between species of different lifestyles such as necrotrophs, hemibiotrophs, or biotrophs is not very strict, or need very specific conditions to manifest. For instance, *Phytophthora infestans* has been placed in all 3 classes (Oliver and Ipcho 2004). Similarly, *Botrytis cinerea* has been placed in different classes as the symptoms it causes differ widely in their severity, depending on the exact interaction with the host (Veloso and Van Kan 2018). Within the genus *Phyllosticta*, some obscurity with respect to lifestyle is present for instance for *P. capitalensis*, which is a widespread endophyte of many hosts including *Citrus* (Wikee *et al.* 2013a), but may cause disease in other hosts such as guava (Arafat 2018). In addition, nonpathogens can evolve a pathogenic lifestyle, and vice-versa, as can be observed with pathogenicity on pea by *Neocosmospora solani*, which is dependent on the presence of only a few genes, or with pathogenicity of *Fusarium oxysporum* on cucurbit species, which is determined by the absence or presence of a mobile pathogenicity chromosome (Temporini and Vanetten 2002; Dong *et al.* 2015; Möller and Stukenbrock 2017; van Dam *et al.* 2017). The possibility for species to be categorized into multiple trophies in the CAZyme-based classification system therefore presents an advantage over the traditional classification system as it allows for a more correct, double classification of species that exhibit multiple lifestyles depending on the host and other environmental parameters.

We compared genomes of species with different lifestyles within the genus *Phyllosticta* specifically, and found several distinctions. We observed that endophytes more often have higher numbers of genes in specific OGs as compared to pathogens. This suggests that the ability to be an endophyte necessitates the presence of additional genes. The ancestral *Dothideomycete* was likely a saprotroph, however, the most common ancestor of the *Botryosphaeriales*, the order in which *Phyllosticta* resides, was probably a plant pathogen, as determined by ancestral state reconstruction (Abdollahzadeh *et al.* 2020; Haridas *et al.* 2020). The evolution of *Phyllosticta* endophytes from *Phyllosticta* pathogens through a gain of genes and thereby gain of abilities is therefore a plausible scenario. With respect to lifestyle definition in *Phyllosticta*, *P. citrichinaensis* is the most ambiguous citrus-related species within the genus. By comparing the genome of this species with those of other species with different lifestyles within the genus *Phyllosticta*, we aimed to elucidate the lifestyle of this species. In several aspects, we found *P. citrichinaensis* to be most similar to endophytes. For instance, *P. citrichinaensis* shares more OGs specific to species of one lifestyle with endophytic species (50) than it does with pathogenic species (30), and none of its BGCs was predicted to produce a squalestatin, which was also the case for all of the endophytes, but not for the pathogens. In addition, CAZyme families that had more genes in endophytes than in pathogens, often also had more genes in *P. citrichinaensis*. Furthermore, growth of *P. citrichinaensis* was not inhibited by the presence of sugar beet pulp, similarly to endophytic species. In contrast, its broader carbon utilization capabilities were more comparable to those of pathogenic species. Another aspect in which *P. citrichinaensis* was comparable to pathogens, was the presence of more putative peroxiredoxin genes in pathogens and *P. citrichinaensis* as compared to endophytes. On other aspects, *P. citrichinaensis* did not compare well with species of either lifestyle, such as the number of OGs that had more genes in species of either lifestyle: in almost a third of the cases, *P. citrichinaensis* did not match the gene numbers of either lifestyle. The number of effector genes that *P. citrichinaensis* shared with species of either lifestyle also suggests an intermediate pattern. The lifestyle of *P. citrichinaensis* cannot be univocally determined without performing pathogenicity assays, but as these are currently not available for this species, the data presented here give a good estimation that shows that *P. citrichinaensis* is an intermediate taxon, not perfectly fitting into any of the currently defined lifestyle definitions.

Research performed in recent years has shown with increasing confidence that borders between lifestyles simply are not very strict and in fact are subject to constant change. Examples such as *B. cinerea* and *Phytophthora infestans*, which both have been placed in multiple lifestyle classes depending on the host and other environmental parameters, demonstrate that our current classification systems are not always adequate to separate species into different lifestyles (Oliver and Ipcho 2004; Veloso and Van Kan 2018). In addition, the ability of species to be pathogenic to a specific host may change with the gain of only a few genes (Temporini and Vanetten 2002; van Dam *et al.* 2017). Classifying plant-associated microbes into different lifestyles is an important area of research as it allows for the identification of genomic parameters that are required for pathogenicity, and therefore aids in the search of a remedy against such pathogens. However, a classification system is only valuable if it allows for the accurate separation of species; an incorrectly classified organism may lead to incorrect conclusions and could cause much confusion. A classification such as the one proposed by Hane *et al.* which is based on the number of CAZyme genes in a species’ genome, is a significant improvement since it allows for overlap between lifestyles. Further development of such classifications for instance by the addition of other genomic parameters such as the presence of effectors could lead to the development of a more accurate and useful classification system in the future.

## Data availability

The whole-genome sequencing data including annotations for the newly sequenced *Phyllosticta citrichinaensis* genome are publicly available at the JGI genome portal: https://mycocosm.jgi.doe.gov/Pcit129764 (last accessed: 22 March 2022). The authors affirm that all other data necessary for confirming the conclusions of the article are present within the article, figures, and (supplementary) tables.

Supplemental material is available at *G3* online.

## Supplementary Material

jkac061_Supplementary_Figure_1

jkac061_Supplementary_Figure_2

jkac061_Supplementary_Table_1

jkac061_Supplementary_Table_2

jkac061_Supplementary_Table_3

jkac061_Supplementary_Table_4

jkac061_Supplementary_Table_5

## References

[jkac061-B1] Abdollahzadeh J , Groenewald JZ, Coetzee MPA, Wingfield MJ, Crous PW. Evolution of lifestyles in Capnodiales. Stud Mycol. 2020;95:381–414. 32855743 10.1016/j.simyco.2020.02.004 PMC7426231

[jkac061-B2] Arafat K. A novel isolate of *Phyllosticta capitalensis* causes black spot disease on guava fruit in Egypt. Asian J Plant Pathol. 2018;12(1):27–37.

[jkac061-B3] Blin K , Shaw S, Steinke K, Villebro R, Ziemert N, Lee SY, Medema MH, Weber T. antiSMASH 5.0: updates to the secondary metabolite genome mining pipeline. Nucleic Acids Res. 2019;47(W1):W81–W87. 31032519 10.1093/nar/gkz310 PMC6602434

[jkac061-B4] Buijs VA , Zuijdgeest XCL, Groenewald JZ, Crous PW, de Vries RP. Carbon utilization and growth-inhibition of citrus-colonizing Phyllosticta species. Fungal Biol. 2021;125(10):815–825. 34537177 10.1016/j.funbio.2021.05.003

[jkac061-B5] Chiapello H , Mallet L, Guérin C, Aguileta G, Amselem J, Kroj T, Ortega-Abboud E, Lebrun M-H, Henrissat B, Gendrault A, et al Deciphering genome content and evolutionary relationships of isolates from the fungus *Magnaporthe oryzae* attacking different host plants. Genome Biol Evol. 2015;7(10):2896–2912. 26454013 10.1093/gbe/evv187 PMC4684704

[jkac061-B6] Conway JR , Lex A, Gehlenborg N. UpSetR: an R package for the visualization of intersecting sets and their properties. Bioinformatics. 2017;33(18):2938–2940. 28645171 10.1093/bioinformatics/btx364 PMC5870712

[jkac061-B7] De Wit PJGM , Mehrabi R, Van den Burg HA, Stergiopoulos I. Fungal effector proteins: past, present and future. Mol Plant Pathol. 2009;10(6):735–747. 19849781 10.1111/j.1364-3703.2009.00591.x PMC6640362

[jkac061-B8] Dong S , Raffaele S, Kamoun S. The two-speed genomes of filamentous pathogens: waltz with plants. Curr Opin Genet Dev. 2015;35:57–65. 26451981 10.1016/j.gde.2015.09.001

[jkac061-B9] Emms DM , Kelly S. OrthoFinder: phylogenetic orthology inference for comparative genomics. Genome Biol. 2019;20(1):238. 31727128 10.1186/s13059-019-1832-y PMC6857279

[jkac061-B10] EFSA PLH Panel (EFSA Panel on Plant Health). Scientific Opinion on the risk of *Phyllosticta citricarpa* (*Guignardia citricarpa*) for the EU territory with identification and evaluation of risk reduction options. EFSA Journal 2014;12(2):3557, 243 pp.

[jkac061-B11] Eustáquio Lanza F , Germano Metzker T, Vinhas T, Behlau F, José Silva Junior G. Critical fungicide spray period for citrus black spot control in São Paulo State, Brazil. Plant Dis. 2018;102(2):334–340. 30673526 10.1094/PDIS-04-17-0537-RE

[jkac061-B12] Fisher MC , Henk DA, Briggs CJ, Brownstein JS, Madoff LC, McCraw SL, Gurr SJ. Emerging fungal threats to animal, plant and ecosystem health. Nature. 2012;484(7393):186–194. 22498624 10.1038/nature10947 PMC3821985

[jkac061-B13] Fouché S , Plissonneau CM, Croll D. The birth and death of effectors in rapidly evolving filamentous pathogen genomes. Curr Opin Microbiol. 2018;46:34–42. 29455143 10.1016/j.mib.2018.01.020

[jkac061-B14] Fudal I , Ross S, Brun H, Besnard A-L, Ermel M, Kuhn M-L, Balesdent M-H, Rouxel T. Repeat-Induced Point Mutation (RIP) as an alternative mechanism of evolution toward virulence in *Leptosphaeria maculans*. Mol Plant Microbe Interact. 2009;22(8):932–941. 19589069 10.1094/MPMI-22-8-0932

[jkac061-B15] Gardiner DM , McDonald MC, Covarelli L, Solomon PS, Rusu AG, Marshall M, Kazan K, Chakraborty S, McDonald BA, Manners JM, et al Comparative pathogenomics reveals horizontally acquired novel virulence genes in fungi infecting cereal hosts. PLoS Pathog. 2012;8(9):e1002952. 23028337 10.1371/journal.ppat.1002952 PMC3460631

[jkac061-B16] Gibriel HAY , Thomma BPHJ, Seidl MF. The age of effectors: genome-based discovery and applications. Phytopathology. 2016;106(10):1206–1212. 27050568 10.1094/PHYTO-02-16-0110-FI

[jkac061-B17] Glienke C , Pereira OL, Stringari D, Fabris J, Kava-Cordeiro V, Galli-Terasawa L, Cunnington J, Shivas RG, Groenewald JZ, Crous PW, et al Endophytic and pathogenic Phyllosticta species, with reference to those associated with citrus black spot. Persoonia. 2011;26:47–56. 22025803 10.3767/003158511X569169 PMC3160796

[jkac061-B18] Grigoriev IV , Nikitin R, Haridas S, Kuo A, Ohm R, Otillar R, Riley R, Salamov A, Zhao X, Korzeniewski F, et al MycoCosm portal: gearing up for 1000 fungal genomes. Nucleic Acids Res. 2014;42:D699–D704. 24297253 10.1093/nar/gkt1183 PMC3965089

[jkac061-B19] Gu Z , Eils R, Schlesner M. Complex heatmaps reveal patterns and correlations in multidimensional genomic data. Bioinformatics. 2016;32(18):2847–2849. 27207943 10.1093/bioinformatics/btw313

[jkac061-B20] Guarnaccia V , Gehrmann T, Silva-Junior GJ, Fourie PH, Haridas S, Vu D, Spatafora J, Martin FM, Robert V, Grigoriev IV, et al *Phyllosticta citricarpa* and sister species of global importance to citrus. Mol Plant Pathol. 2019;20(12):1619–1635. 31512371 10.1111/mpp.12861 PMC6859488

[jkac061-B21] Guarnaccia V , Groenewald JZ, Li H, Glienke C, Carstens E, Hattingh V, Fourie PH, Crous PW. First report of *Phyllosticta citricarpa* and description of two new species, *P. paracapitalensis* and *P. paracitricarpa*, from citrus in Europe. Stud Mycol. 2017;87:161–185. 28720979 10.1016/j.simyco.2017.05.003 PMC5502700

[jkac061-B22] Gurevich A , Saveliev V, Vyahhi N, Tesler G. QUAST: quality assessment tool for genome assemblies. Bioinformatics. 2013;29(8):1072–1075. 23422339 10.1093/bioinformatics/btt086 PMC3624806

[jkac061-B23] Hane JK , Paxman J, Jones DAB, Oliver RP, de Wit P. “CATAStrophy,” a genome-informed trophic classification of filamentous plant pathogens—how many different types of filamentous plant pathogens are there? Front Microbiol. 2020;10:3088. 32038539 10.3389/fmicb.2019.03088 PMC6986263

[jkac061-B24] Haridas S , Albert R, Binder M, Bloem J, LaButti K, Salamov A, Andreopoulos B, Baker SE, Barry K, Bills G, et al 101 Dothideomycetes genomes: a test case for predicting lifestyles and emergence of pathogens. Stud Mycol. 2020;96:141–153. 32206138 10.1016/j.simyco.2020.01.003 PMC7082219

[jkac061-B25] Hegedus DD , Rimmer SR. *Sclerotinia sclerotiorum*: when “to be or not to be” a pathogen? FEMS Microbiol Lett. 2005;251(2):177–184. 16112822 10.1016/j.femsle.2005.07.040

[jkac061-B26] Hüttel W , Müller M. Regio- and stereoselective intermolecular phenol coupling enzymes in secondary metabolite biosynthesis. Nat Prod Rep. 2021;38(5):1011–1043. 33196733 10.1039/d0np00010h

[jkac061-B27] Jones DAB , Rozano L, Debler JW, Mancera RL, Moolhuijzen PM, Hane JK An automated and combinative method for the predictive ranking of candidate effector proteins of fungal plant pathogens. Sci Reports. 2021;11:19731. 10.1038/s41598-021-99363-0 PMC8492765 34611252

[jkac061-B28] Kabbage M , Yarden O, Dickman MB. Pathogenic attributes of *Sclerotinia sclerotiorum*: switching from a biotrophic to necrotrophic lifestyle. Plant Sci. 2015;233:53–60. 25711813 10.1016/j.plantsci.2014.12.018

[jkac061-B29] Kim K-T , Jeon J, Choi J, Cheong K, Song H, Choi G, Kang S, Lee Y-H. Kingdom-wide analysis of fungal Small Secreted Proteins (SSPs) reveals their potential role in host association. Front Plant Sci. 2016;7:186. 26925088 10.3389/fpls.2016.00186 PMC4759460

[jkac061-B30] King BC , Waxman KD, Nenni NV, Walker LP, Bergstrom GC, Gibson DM. Arsenal of plant cell wall degrading enzymes reflects host preference among plant pathogenic fungi. Biotechnol. Biofuels. 2011;4:4–14. 21324176 10.1186/1754-6834-4-4 PMC3051899

[jkac061-B31] Klosterman SJ , Subbarao KV, Kang S, Veronese P, Gold SE, Thomma BPHJ, Chen Z, Henrissat B, Lee Y-H, Park J, et al Comparative genomics yields insights into niche adaptation of plant vascular wilt pathogens. PLoS Pathog. 2011;7(7):e1002137. 21829347 10.1371/journal.ppat.1002137 PMC3145793

[jkac061-B32] Kotzé JM. Black spot. In: Compendium of Citrus Diseases. American Phytopathological Society Press Inc. Timmer LW, Garnsey SM, Graham JH, editors. MN, USA: The American Phytopathological Society; 2000. p. 23–25.

[jkac061-B33] Kubicek CP , Starr TL, Glass NL. Plant cell wall–degrading enzymes and their secretion in plant-pathogenic fungi. Annu Rev Phytopathol. 2014;52:427–451. 25001456 10.1146/annurev-phyto-102313-045831

[jkac061-B34] Kubicek CP , Steindorff AS, Chenthamara K, Manganiello G, Henrissat B, Zhang J, Cai F, Kopchinskiy AG, Kubicek EM, Kuo A, et al Evolution and comparative genomics of the most common Trichoderma species. BMC Genomics. 2019;20(1):1–24. 31189469 10.1186/s12864-019-5680-7 PMC6560777

[jkac061-B35] Lindo L , Cardoza RE, Lorenzana A, Casquero PA, Gutiérrez S. Identification of plant genes putatively involved in the perception of fungal ergosterol-squalene. J Integr Plant Biol. 2020;62(7):927–947. 31436383 10.1111/jipb.12862 PMC7383801

[jkac061-B36] Lombard V , Golaconda Ramulu H, Drula E, Coutinho PM, Henrissat B. The carbohydrate-active enzymes database (CAZy) in 2013. Nucleic Acids Res. 2014;42(Database issue):D490–D495. 24270786 10.1093/nar/gkt1178 PMC3965031

[jkac061-B37] Manni M , Berkeley MR, Seppey M, Simão FA, Zdobnov EM. BUSCO update: novel and streamlined workflows along with broader and deeper phylogenetic coverage for scoring of eukaryotic, prokaryotic, and viral genomes. Mol Biol Evol. 2021;38(10):4647–4654. 34320186 10.1093/molbev/msab199 PMC8476166

[jkac061-B38] Marçais G , Delcher AL, Phillippy AM, Coston R, Salzberg SL, Zimin A. MUMmer4: a fast and versatile genome alignment system. PLoS Comput Biol. 2018;14(1):e1005944. 29373581 10.1371/journal.pcbi.1005944 PMC5802927

[jkac061-B39] Mir AA , Park S-Y, Sadat MA, Kim S, Choi J, Jeon J, Lee Y-H. Systematic characterization of the peroxidase gene family provides new insights into fungal pathogenicity in *Magnaporthe oryzae*. Sci Rep. 2015;5:1–14. 10.1038/srep11831 PMC4488832 26134974

[jkac061-B40] Möller M , Stukenbrock EH. Evolution and genome architecture in fungal plant pathogens. Nat Rev Microbiol. 2017;15(12):756–771. 29123226 10.1038/nrmicro.2017.143

[jkac061-B41] O’Connell RJ , Thon MR, Hacquard S, Amyotte SG, Kleemann J, Torres MF, Damm U, Buiate EA, Epstein L, Alkan N, et al Lifestyle transitions in plant pathogenic Colletotrichum fungi deciphered by genome and transcriptome analyses. Nat Genet. 2012;44:1060–1065. 22885923 10.1038/ng.2372 PMC9754331

[jkac061-B42] Ohm RA , Feau N, Henrissat B, Schoch CL, Horwitz BA, Barry KW, Condon BJ, Copeland AC, Dhillon B, Glaser F, et al Diverse lifestyles and strategies of plant pathogenesis encoded in the genomes of eighteen dothideomycetes fungi (A. Andrianopoulos, Ed.). PLoS Pathog. 2012;8(12):e1003037. 23236275 10.1371/journal.ppat.1003037 PMC3516569

[jkac061-B43] Oliver RP , Ipcho SVS. Arabidopsis pathology breathes new life into the necrotrophs-vs.-biotrophs classification of fungal pathogens. Mol Plant Pathol. 2004;5(4):347–352. 20565602 10.1111/j.1364-3703.2004.00228.x

[jkac061-B44] Petters-Vandresen DAL , Rossi BJ, Groenewald JZ, Crous PW, Machado MA, Stukenbrock EH, Glienke C. Mating-type locus rearrangements and shifts in thallism states in citrus-associated Phyllosticta species. Fungal Genet Biol. 2020;144:103444. 32822858 10.1016/j.fgb.2020.103444

[jkac061-B45] Plissonneau C , Benevenuto J, Mohd-Assaad N, Fouché S, Hartmann FE, Croll D. Using population and comparative genomics to understand the genetic basis of effector-driven fungal pathogen evolution. Front Plant Sci. 2017;8:119. 28217138 10.3389/fpls.2017.00119 PMC5289978

[jkac061-B46] Lo Presti L , Lanver D, Schweizer G, Tanaka S, Liang L, Tollot M, Zuccaro A, Reissmann S, Kahmann R. Fungal effectors and plant susceptibility. Annu Rev Plant Biol. 2015;66:513–545. 25923844 10.1146/annurev-arplant-043014-114623

[jkac061-B47] Rocha MC , de Godoy KF, Bannitz-Fernandes R, Fabri JHTM, Barbosa MMF, de Castro PA, Almeida F, Goldman GH, da Cunha AF, Netto LES, et al Analyses of the three 1-Cys Peroxiredoxins from *Aspergillus fumigatus* reveal that cytosolic Prx1 is central to H_2_O_2_ metabolism and virulence. Sci Reports. 2018;18:12314. 10.1038/s41598-018-30108-2 PMC6098058 30120327

[jkac061-B48] Rodrigues CM , Takita MA, Silva NV, Ribeiro-Alves M, Machado MA. Comparative genome analysis of *Phyllosticta citricarpa* and *Phyllosticta capitalensis*, two fungi species that share the same host. BMC Genomics. 2019;20(1):554. 31277573 10.1186/s12864-019-5911-y PMC6612112

[jkac061-B49] Rovenich H , Boshoven JC, Thomma BPHJ. Filamentous pathogen effector functions of pathogens, hosts and microbiomes. Curr Opin Plant Biol. 2014;20:96–103. 24879450 10.1016/j.pbi.2014.05.001

[jkac061-B50] Schwelm A , Bradshaw RE. Genetics of Dothistromin biosynthesis of *Dothistroma septosporum*: an update. Toxins (Basel). 2010;2(11):2680–2698. 22069571 10.3390/toxins2112680 PMC3153176

[jkac061-B51] Shin JY , Bui D-C, Lee Y, Nam H, Jung S, Fang M, Kim J-C, Lee T, Kim H, Choi GJ, et al Functional characterization of cytochrome P450 monooxygenases in the cereal head blight fungus *Fusarium graminearum*. Environ Microbiol. 2017;19(5):2053–2067. 28296081 10.1111/1462-2920.13730

[jkac061-B52] Siewers V , Viaud M, Jimenez-Teja D, Collado IG, Gronover CS, Pradier J-M, Tudzynski B, Tudzynski P. Functional analysis of the cytochrome P450 monooxygenase gene bcbot1 of *Botrytis cinerea* indicates that botrydial is a strain-specific virulence factor. Mol Plant Microbe Interact. 2005;18(6):602–612. 15986930 10.1094/MPMI-18-0602

[jkac061-B53] Singh RP , Hodson DP, Huerta-Espino J, Jin Y, Bhavani S, Njau P, Herrera-Foessel S, Singh PK, Singh S, Govindan V, et al The emergence of Ug99 races of the stem rust fungus is a threat to world wheat production. Annu Rev Phytopathol. 2011;49:465–481. 21568701 10.1146/annurev-phyto-072910-095423

[jkac061-B54] Spanu PD. The genomics of obligate (and nonobligate) biotrophs. Annu Rev Phytopathol. 2012;50:91–109. 22559067 10.1146/annurev-phyto-081211-173024

[jkac061-B55] Sperschneider J , Dodds PN, Gardiner DM, Manners JM, Singh KB, Taylor JM. Advances and challenges in computational prediction of effectors from plant pathogenic fungi. PLoS Pathog. 2015;11(5):e1004806. 26020524 10.1371/journal.ppat.1004806 PMC4447458

[jkac061-B56] Stergiopoulos I , Kourmpetis YAI, Slot JC, Bakker FT, De Wit PJGM, Rokas A. In silico characterization and molecular evolutionary analysis of a novel superfamily of fungal effector proteins. Mol Biol Evol. 2012;29(11):3371–3384. 22628532 10.1093/molbev/mss143

[jkac061-B57] Temporini ED , Vanetten HD. Distribution of the pea pathogenicity (PEP) genes in the fungus *Nectria haematococca* mating population VI. Curr Genet. 2002;41(2):107–114. 12073092 10.1007/s00294-002-0279-x

[jkac061-B58] ten Have A , Tenberge KB, Benen JAE, Tudzynski P, Visser J, van Kan JAL. The contribution of cell wall degrading enzymes to pathogenesis of fungal plant pathogens. In: Kempken F, editor. The Mycota XI: agricultural Applications. Berlin, Heidelberg: Springer Berlin Heidelberg; 2002. p. 341–358.

[jkac061-B59] van Dam P , Fokkens L, Ayukawa Y, van der Gragt M, Ter Horst A, Brankovics B, Houterman PM, Arie T, Rep M. A mobile pathogenicity chromosome in *Fusarium oxysporum* for infection of multiple cucurbit species. Sci Rep. 2017;7(1):9042. 28831051 10.1038/s41598-017-07995-y PMC5567276

[jkac061-B60] van Dam P , Rep M. The distribution of miniature Impala elements and six genes in the Fusarium genus is suggestive of horizontal gene transfer. J Mol Evol. 2017;85(1–2):14–25. 28744785 10.1007/s00239-017-9801-0 PMC5579170

[jkac061-B61] van den Brink J , de Vries RP. Fungal enzyme sets for plant polysaccharide degradation. Appl Microbiol Biotechnol. 2011;91(6):1477–1492. 21785931 10.1007/s00253-011-3473-2 PMC3160556

[jkac061-B62] Veloso J , Van Kan JAL. Many shades of grey in Botrytis-host plant interactions. Trends Plant Sci. 2018;23(7):613–622. 29724660 10.1016/j.tplants.2018.03.016

[jkac061-B63] Wang M , Liu B, Ruan R, Zeng Y, Luo J, Li H. Genomic sequencing of *Phyllosticta citriasiana* provides insight into its conservation and diversification with two closely related Phyllosticta species associated with citrus. Front Microbiol. 2020;10:2979. 31998266 10.3389/fmicb.2019.02979 PMC6965161

[jkac061-B64] Wang X , Chen G, Huang F, Zhang J, Hyde KD, Li H. Phyllosticta species associated with citrus diseases in China. Fungal Divers. 2012;52(1):209–224.

[jkac061-B65] Wikee S , Lombard L, Crous PW, Nakashima C, Motohashi K, Chukeatirote E, Alias SA, McKenzie EHC, Hyde KD. *Phyllosticta capitalensis*, a widespread endophyte of plants. Fungal Divers. 2013a;60(1):91–105.

[jkac061-B66] Wikee S , Lombard L, Nakashima C, Motohashi K, Chukeatirote E, Cheewangkoon R, McKenzie EHC, Hyde KD, Crous PW. A phylogenetic re-evaluation of *Phyllosticta* (Botryosphaeriales). Stud Mycol. 2013b;76(1):1–29. 24302788 10.3114/sim0019 PMC3825230

[jkac061-B67] Wulandari NF , To-anun C, Hyde KD, Duong LM, de Gruyter J. *Phyllosticta citriasiana* sp. nov., the cause of Citrus tan spot of *Citrus maxima* in Asia. Fungal Divers. 2009;34:23–39.

[jkac061-B68] Yang Y , Zhang Y, Li B, Yang X, Dong Y, Qiu D. A *Verticillium dahliae* pectate lyase induces plant immune responses and contributes to virulence. Front Plant Sci. 2018;9:1271. 30271415 10.3389/fpls.2018.01271 PMC6146025

[jkac061-B69] Zhang H , Yohe T, Huang L, Entwistle S, Wu P, Yang Z, Busk PK, Xu Y, Yin Y. dbCAN2: a meta server for automated carbohydrate-active enzyme annotation. Nucleic Acids Res. 2018;46(W1):W95–W101. 29771380 10.1093/nar/gky418 PMC6031026

