# Supplementary figures and images for: Enemy or ally: a genomic approach to elucidate the lifestyle of Phyllosticta citrichinaensis
Source: G3 (Bethesda). 2022 Mar 21;12(5):jkac061. doi: 10.1093/g3journal/jkac061 (PMC9073689; doi:10.1093/g3journal/jkac061)

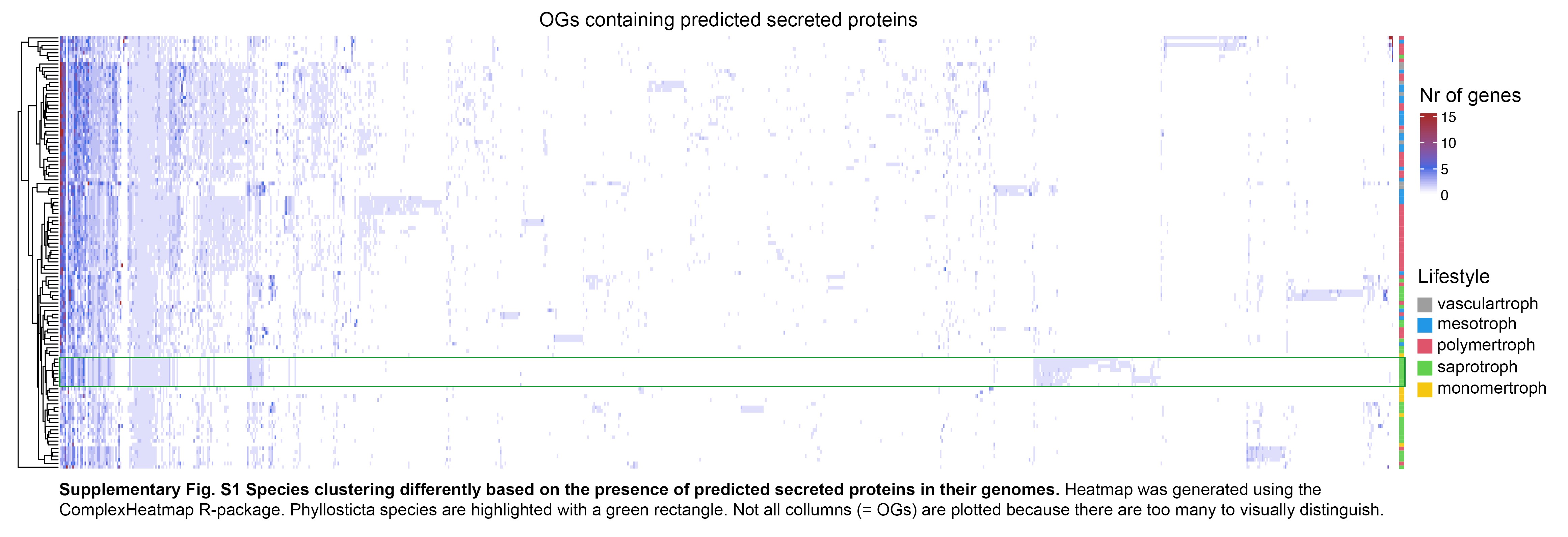

Supplement: jkac061_Supplementary_Figure_1 [file jkac061_supplementary_figure_1.jpeg]

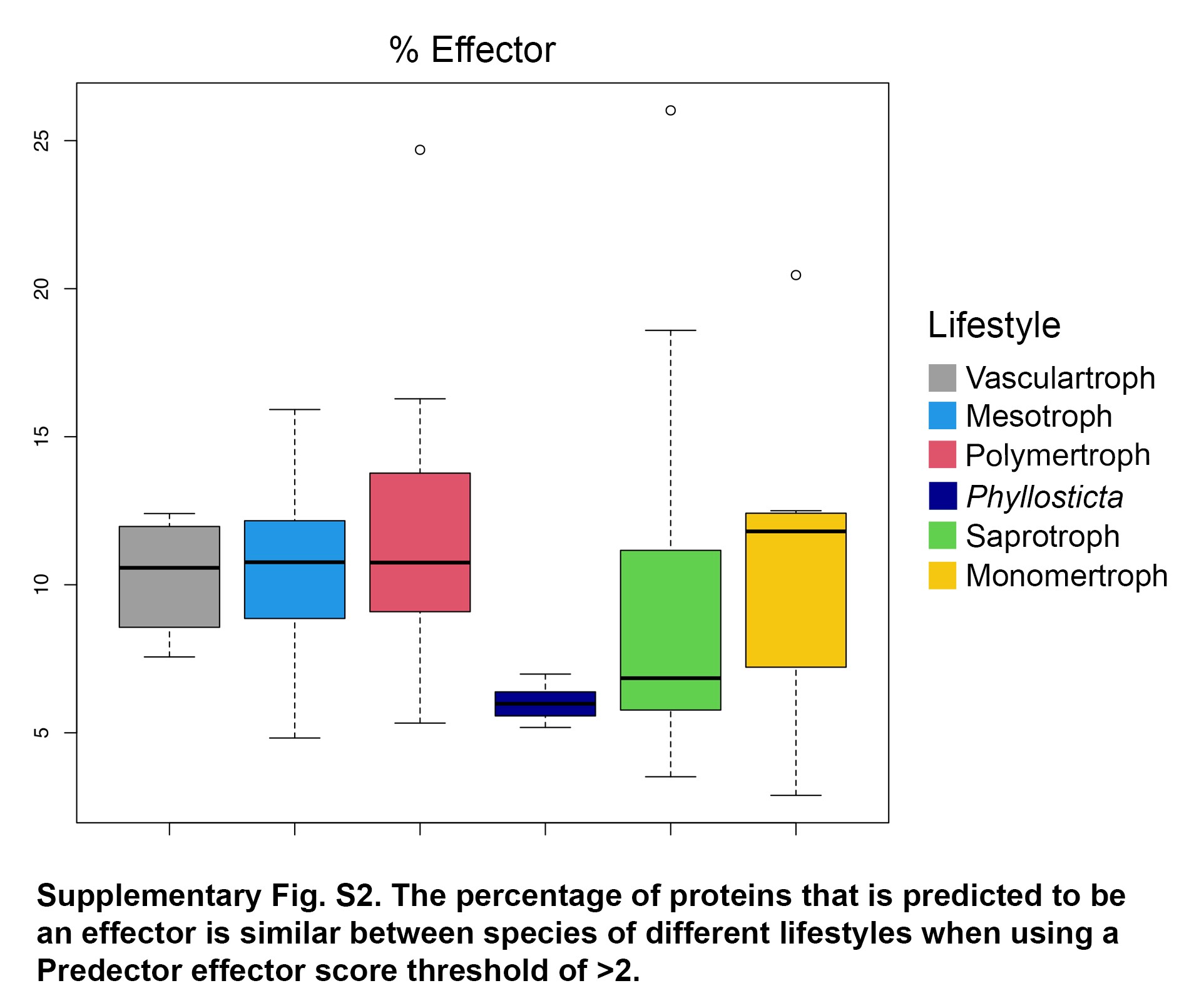

Supplement: jkac061_Supplementary_Figure_2 [file jkac061_supplementary_figure_2.jpeg]
